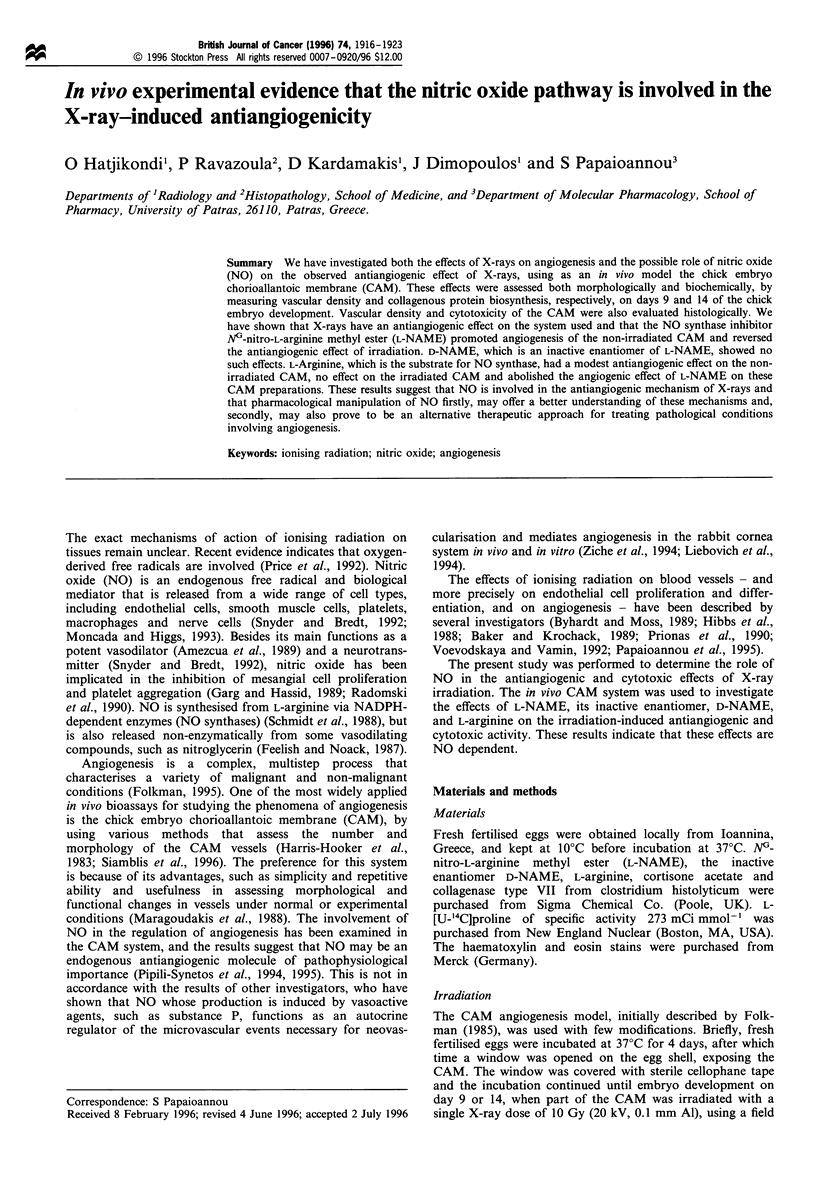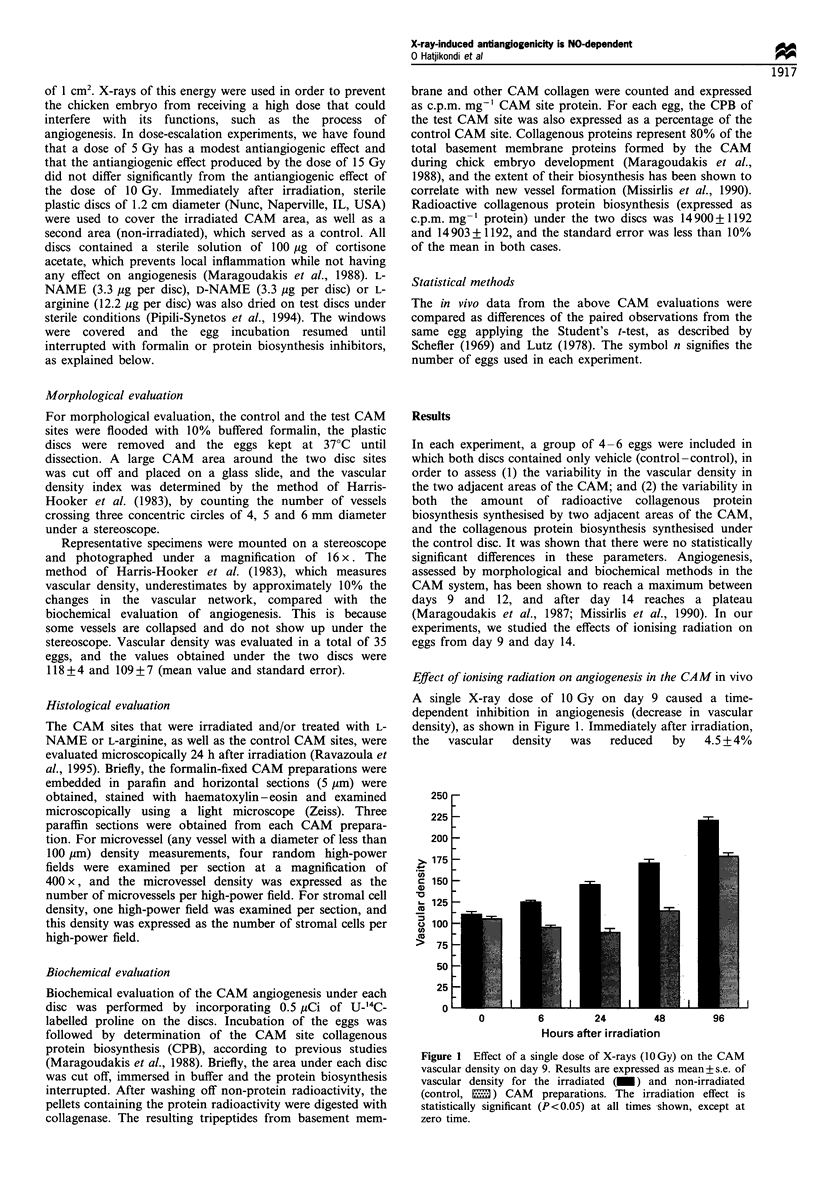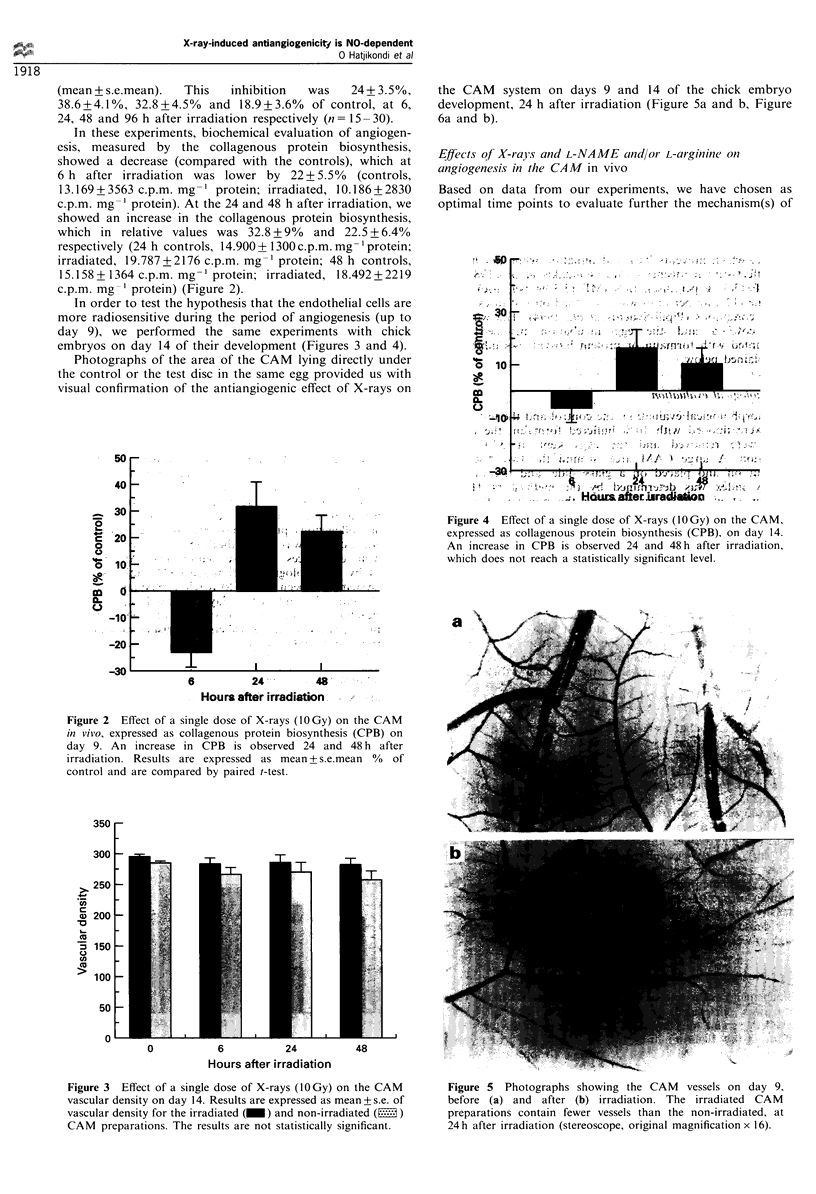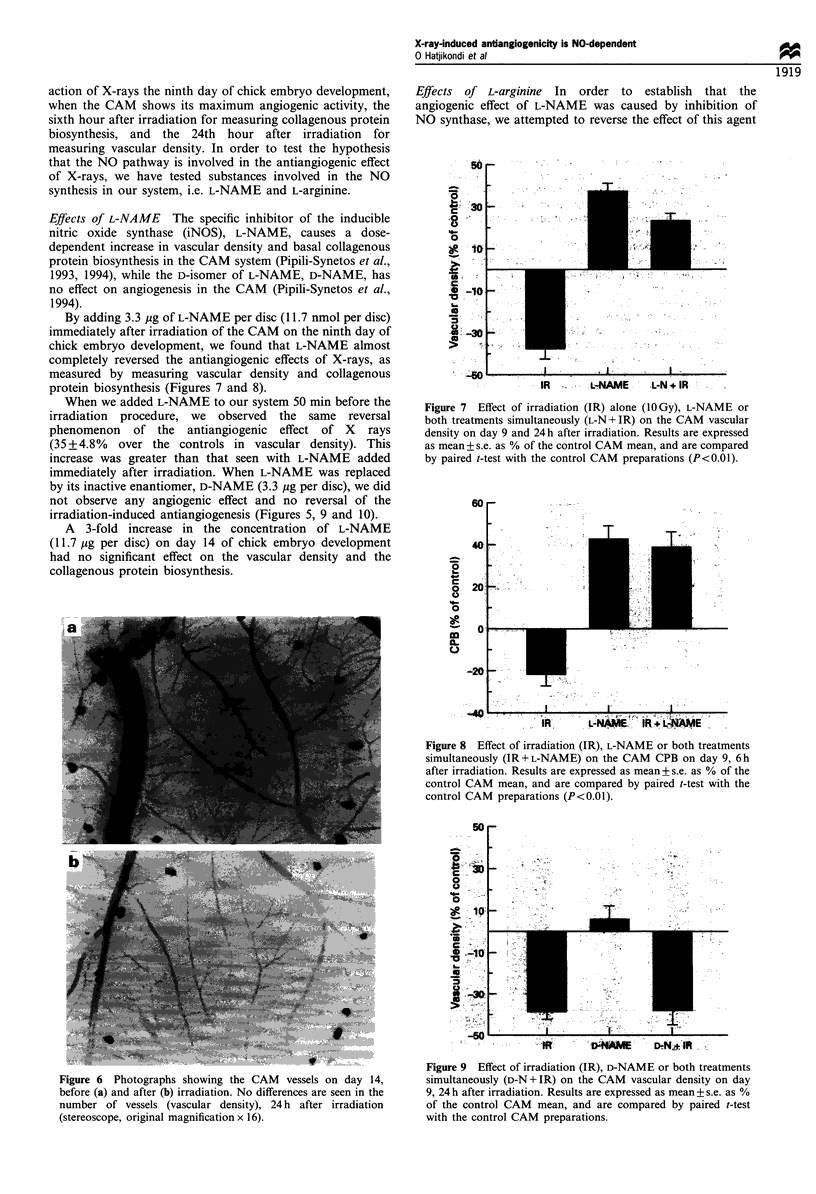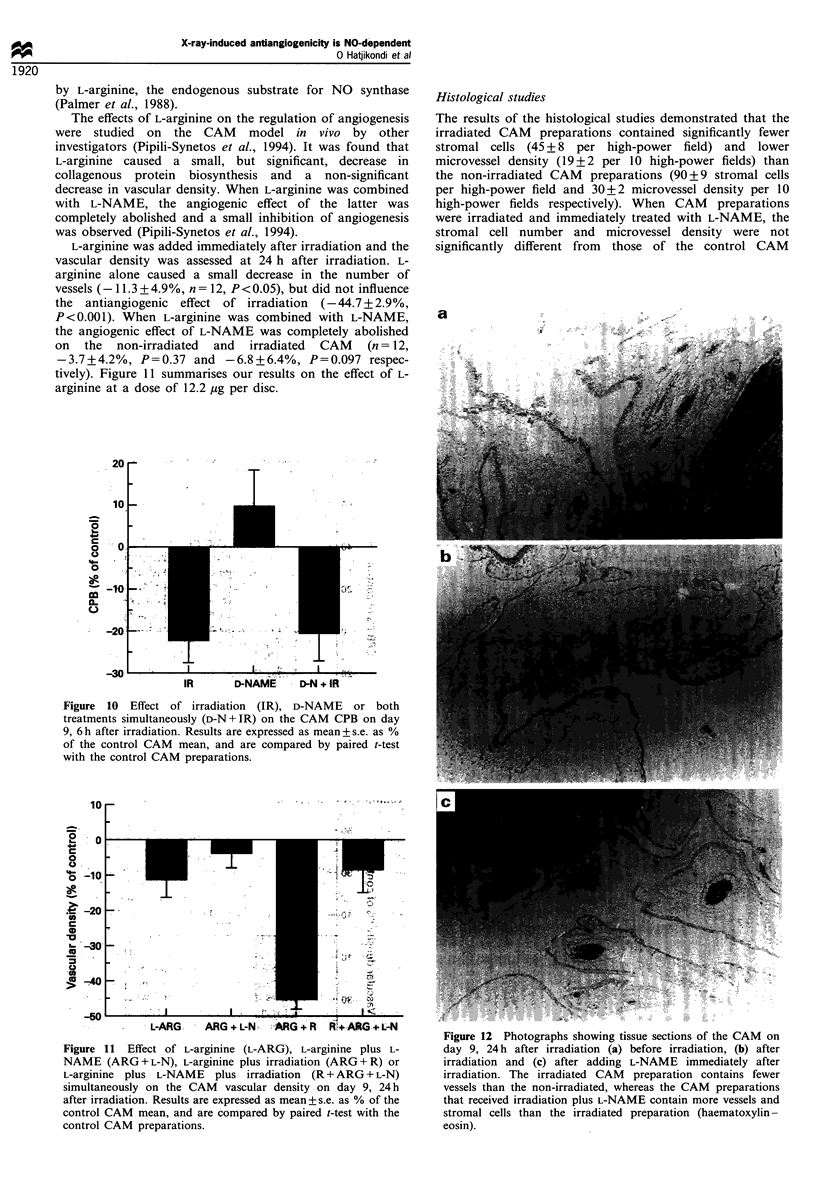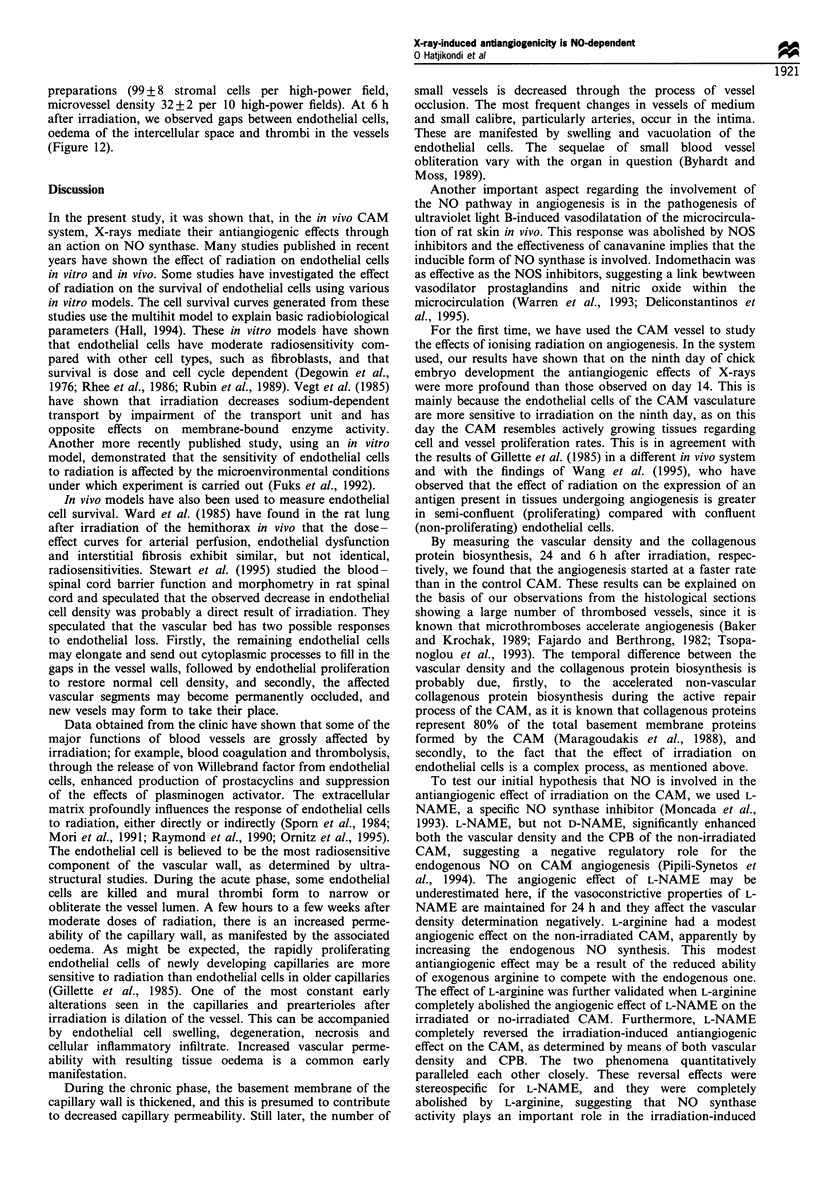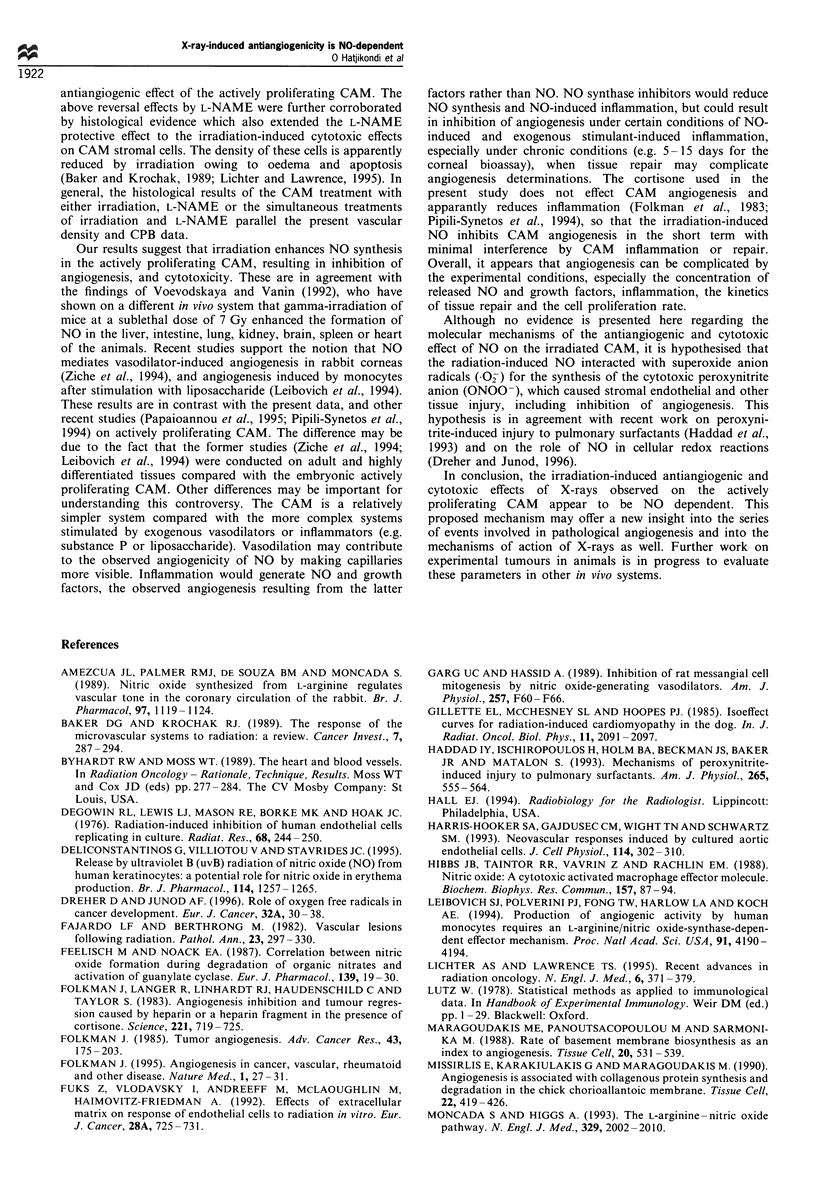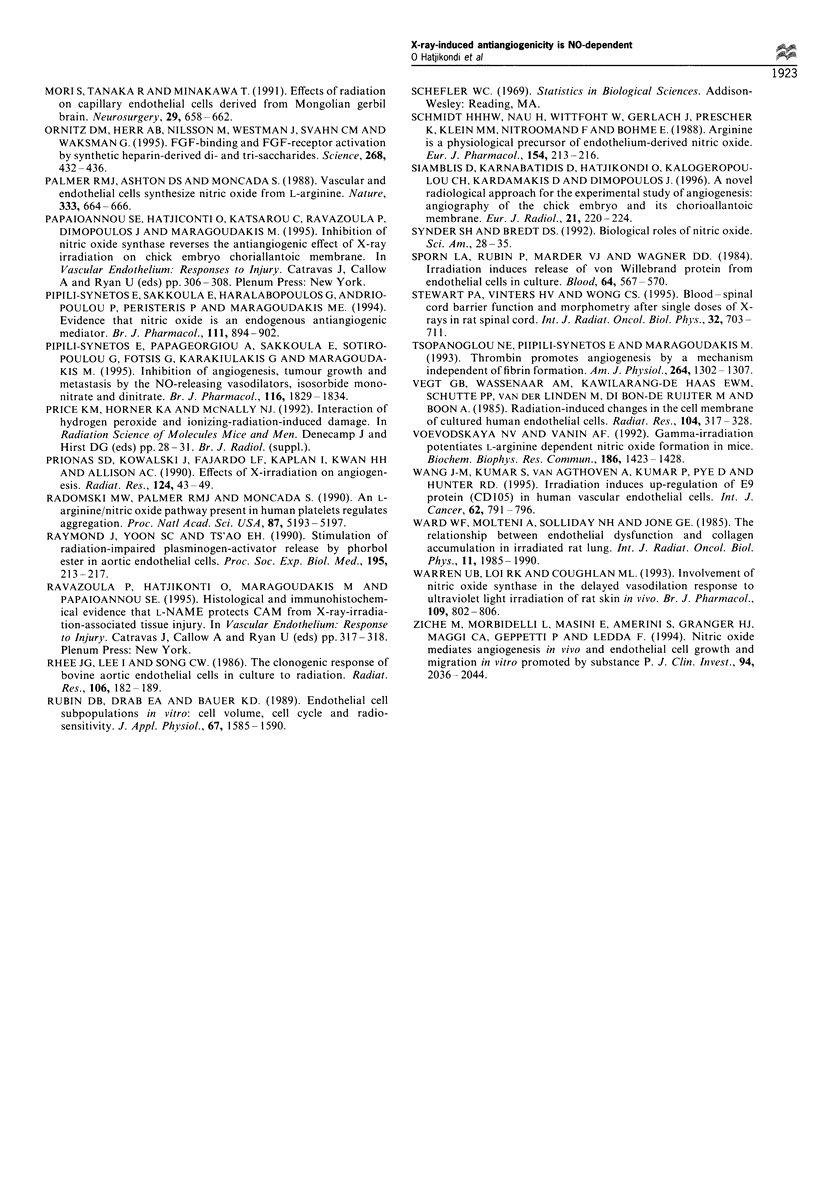# In vivo experimental evidence that the nitric oxide pathway is involved in the X-ray-induced antiangiogenicity.

**DOI:** 10.1038/bjc.1996.653

**Published:** 1996-12

**Authors:** O. Hatjikondi, P. Ravazoula, D. Kardamakis, J. Dimopoulos, S. Papaioannou

**Affiliations:** Department of Radiology, School of Medicine, University of Patras, Greece.

## Abstract

**Images:**


British Journal of Cancer (1996) 74, 1916-1923
© 1996 Stockton Press All rights reserved 0007-0920/96 $12.00

### *In vivo* experimental evidence that the nitric oxide pathway is involved in the X-ray–induced antiangiogenicity

O Hatjikondi[1], P Ravazoula[2], D Kardamakis[1], J Dimopoulos[1] and S Papaioannou[3]

*Departments of [1]Radiology and [2]Histopathology, School of Medicine, and [3]Department of Molecular Pharmacology, School of Pharmacy, University of Patras, 26110, Patras, Greece.*

**Summary** We have investigated both the effects of X-rays on angiogenesis and the possible role of nitric oxide (NO) on the observed antiangiogenic effect of X-rays, using as an *in vivo* model the chick embryo chorioallantoic membrane (CAM). These effects were assessed both morphologically and biochemically, by measuring vascular density and collagenous protein biosynthesis, respectively, on days 9 and 14 of the chick embryo development. Vascular density and cytotoxicity of the CAM were also evaluated histologically. We have shown that X-rays have an antiangiogenic effect on the system used and that the NO synthase inhibitor $N^G$-nitro-L-arginine methyl ester (L-NAME) promoted angiogenesis of the non-irradiated CAM and reversed the antiangiogenic effect of irradiation. D-NAME, which is an inactive enantiomer of L-NAME, showed no such effects. L-Arginine, which is the substrate for NO synthase, had a modest antiangiogenic effect on the non-irradiated CAM, no effect on the irradiated CAM and abolished the angiogenic effect of L-NAME on these CAM preparations. These results suggest that NO is involved in the antiangiogenic mechanism of X-rays and that pharmacological manipulation of NO firstly, may offer a better understanding of these mechanisms and, secondly, may also prove to be an alternative therapeutic approach for treating pathological conditions involving angiogenesis.

**Keywords:** ionising radiation; nitric oxide; angiogenesis

The exact mechanisms of action of ionising radiation on tissues remain unclear. Recent evidence indicates that oxygen-derived free radicals are involved (Price *et al.*, 1992). Nitric oxide (NO) is an endogenous free radical and biological mediator that is released from a wide range of cell types, including endothelial cells, smooth muscle cells, platelets, macrophages and nerve cells (Snyder and Bredt, 1992; Moncada and Higgs, 1993). Besides its main functions as a potent vasodilator (Amezcua *et al.*, 1989) and a neurotransmitter (Snyder and Bredt, 1992), nitric oxide has been implicated in the inhibition of mesangial cell proliferation and platelet aggregation (Garg and Hassid, 1989; Radomski *et al.*, 1990). NO is synthesised from L-arginine via NADPH-dependent enzymes (NO synthases) (Schmidt *et al.*, 1988), but is also released non-enzymatically from some vasodilating compounds, such as nitroglycerin (Feelish and Noack, 1987).

Angiogenesis is a complex, multistep process that characterises a variety of malignant and non-malignant conditions (Folkman, 1995). One of the most widely applied *in vivo* bioassays for studying the phenomena of angiogenesis is the chick embryo chorioallantoic membrane (CAM), by using various methods that assess the number and morphology of the CAM vessels (Harris-Hooker *et al.*, 1983; Siamblis *et al.*, 1996). The preference for this system is because of its advantages, such as simplicity and repetitive ability and usefulness in assessing morphological and functional changes in vessels under normal or experimental conditions (Maragoudakis *et al.*, 1988). The involvement of NO in the regulation of angiogenesis has been examined in the CAM system, and the results suggest that NO may be an endogenous antiangiogenic molecule of pathophysiological importance (Pipili-Synetos *et al.*, 1994, 1995). This is not in accordance with the results of other investigators, who have shown that NO whose production is induced by vasoactive agents, such as substance P, functions as an autocrine regulator of the microvascular events necessary for neovascularisation and mediates angiogenesis in the rabbit cornea system *in vivo* and *in vitro* (Ziche *et al.*, 1994; Liebovich *et al.*, 1994).

The effects of ionising radiation on blood vessels – and more precisely on endothelial cell proliferation and differentiation, and on angiogenesis – have been described by several investigators (Byhardt and Moss, 1989; Hibbs *et al.*, 1988; Baker and Krochack, 1989; Prionas *et al.*, 1990; Voevodskaya and Vamin, 1992; Papaioannou *et al.*, 1995).

The present study was performed to determine the role of NO in the antiangiogenic and cytotoxic effects of X-ray irradiation. The *in vivo* CAM system was used to investigate the effects of L-NAME, its inactive enantiomer, D-NAME, and L-arginine on the irradiation-induced antiangiogenic and cytotoxic activity. These results indicate that these effects are NO dependent.

### Materials and methods

### Materials

Fresh fertilised eggs were obtained locally from Ioannina, Greece, and kept at 10°C before incubation at 37°C. $N^G$-nitro-L-arginine methyl ester (L-NAME), the inactive enantiomer D-NAME, L-arginine, cortisone acetate and collagenase type VII from clostridium histolyticum were purchased from Sigma Chemical Co. (Poole, UK). L-[U-$^{14}$C]proline of specific activity 273 mCi $mmol^{-1}$ was purchased from New England Nuclear (Boston, MA, USA). The haematoxylin and eosin stains were purchased from Merck (Germany).

### Irradiation

The CAM angiogenesis model, initially described by Folkman (1985), was used with few modifications. Briefly, fresh fertilised eggs were incubated at 37°C for 4 days, after which time a window was opened on the egg shell, exposing the CAM. The window was covered with sterile cellophane tape and the incubation continued until embryo development on day 9 or 14, when part of the CAM was irradiated with a single X-ray dose of 10 Gy (20 kV, 0.1 mm Al), using a field

Correspondence: S Papaioannou
Received 8 February 1996; revised 4 June 1996; accepted 2 July 1996

of 1 cm$^2$. X-rays of this energy were used in order to prevent the chicken embryo from receiving a high dose that could interfere with its functions, such as the process of angiogenesis. In dose-escalation experiments, we have found that a dose of 5 Gy has a modest antiangiogenic effect and that the antiangiogenic effect produced by the dose of 15 Gy did not differ significantly from the antiangiogenic effect of the dose of 10 Gy. Immediately after irradiation, sterile plastic discs of 1.2 cm diameter (Nunc, Naperville, IL, USA) were used to cover the irradiated CAM area, as well as a second area (non-irradiated), which served as a control. All discs contained a sterile solution of 100 $\mu$g of cortisone acetate, which prevents local inflammation while not having any effect on angiogenesis (Maragoudakis *et al*., 1988). L-NAME (3.3 $\mu$g per disc), D-NAME (3.3 $\mu$g per disc) or L-arginine (12.2 $\mu$g per disc) was also dried on test discs under sterile conditions (Pipili-Synetos *et al*., 1994). The windows were covered and the egg incubation resumed until interrupted with formalin or protein biosynthesis inhibitors, as explained below.

### *Morphological evaluation*

For morphological evaluation, the control and the test CAM sites were flooded with 10% buffered formalin, the plastic discs were removed and the eggs kept at 37°C until dissection. A large CAM area around the two disc sites was cut off and placed on a glass slide, and the vascular density index was determined by the method of Harris-Hooker *et al.* (1983), by counting the number of vessels crossing three concentric circles of 4, 5 and 6 mm diameter under a stereoscope.

Representative specimens were mounted on a stereoscope and photographed under a magnification of 16×. The method of Harris-Hooker *et al.* (1983), which measures vascular density, underestimates by approximately 10% the changes in the vascular network, compared with the biochemical evaluation of angiogenesis. This is because some vessels are collapsed and do not show up under the stereoscope. Vascular density was evaluated in a total of 35 eggs, and the values obtained under the two discs were 118±4 and 109±7 (mean value and standard error).

### *Histological evaluation*

The CAM sites that were irradiated and/or treated with L-NAME or L-arginine, as well as the control CAM sites, were evaluated microscopically 24 h after irradiation (Ravazoula *et al*., 1995). Briefly, the formalin-fixed CAM preparations were embedded in parafin and horizontal sections (5 $\mu$m) were obtained, stained with haematoxylin–eosin and examined microscopically using a light microscope (Zeiss). Three paraffin sections were obtained from each CAM preparation. For microvessel (any vessel with a diameter of less than 100 $\mu$m) density measurements, four random high-power fields were examined per section at a magnification of 400×, and the microvessel density was expressed as the number of microvessels per high-power field. For stromal cell density, one high-power field was examined per section, and this density was expressed as the number of stromal cells per high-power field.

### *Biochemical evaluation*

Biochemical evaluation of the CAM angiogenesis under each disc was performed by incorporating 0.5 $\mu$Ci of U-$^{14}$C-labelled proline on the discs. Incubation of the eggs was followed by determination of the CAM site collagenous protein biosynthesis (CPB), according to previous studies (Maragoudakis *et al*., 1988). Briefly, the area under each disc was cut off, immersed in buffer and the protein biosynthesis interrupted. After washing off non-protein radioactivity, the pellets containing the protein radioactivity were digested with collagenase. The resulting tripeptides from basement membrane and other CAM collagen were counted and expressed as c.p.m. mg$^{-1}$ CAM site protein. For each egg, the CPB of the test CAM site was also expressed as a percentage of the control CAM site. Collagenous proteins represent 80% of the total basement membrane proteins formed by the CAM during chick embryo development (Maragoudakis *et al*., 1988), and the extent of their biosynthesis has been shown to correlate with new vessel formation (Missirlis *et al*., 1990). Radioactive collagenous protein biosynthesis (expressed as c.p.m. mg$^{-1}$ protein) under the two discs was 14 900±1192 and 14 903±1192, and the standard error was less than 10% of the mean in both cases.

### *Statistical methods*

The *in vivo* data from the above CAM evaluations were compared as differences of the paired observations from the same egg applying the Student's *t*-test, as described by Schefler (1969) and Lutz (1978). The symbol *n* signifies the number of eggs used in each experiment.

### Results

In each experiment, a group of 4–6 eggs were included in which both discs contained only vehicle (control–control), in order to assess (1) the variability in the vascular density in the two adjacent areas of the CAM; and (2) the variability in both the amount of radioactive collagenous protein biosynthesis synthesised by two adjacent areas of the CAM, and the collagenous protein biosynthesis synthesised under the control disc. It was shown that there were no statistically significant differences in these parameters. Angiogenesis, assessed by morphological and biochemical methods in the CAM system, has been shown to reach a maximum between days 9 and 12, and after day 14 reaches a plateau (Maragoudakis *et al*., 1987; Missirlis *et al*., 1990). In our experiments, we studied the effects of ionising radiation on eggs from day 9 and day 14.

### *Effect of ionising radiation on angiogenesis in the CAM* in vivo

A single X-ray dose of 10 Gy on day 9 caused a time-dependent inhibition in angiogenesis (decrease in vascular density), as shown in Figure 1. Immediately after irradiation, the vascular density was reduced by 4.5±4%

**Figure 1** Effect of a single dose of X-rays (10 Gy) on the CAM vascular density on day 9. Results are expressed as mean±s.e. of vascular density for the irradiated (■) and non-irradiated (control, ▒) CAM preparations. The irradiation effect is statistically significant ($P<0.05$) at all times shown, except at zero time.

(mean ± s.e.mean). This inhibition was 24 ± 3.5%, 38.6 ± 4.1%, 32.8 ± 4.5% and 18.9 ± 3.6% of control, at 6, 24, 48 and 96 h after irradiation respectively ($n = 15-30$).

In these experiments, biochemical evaluation of angiogenesis, measured by the collagenous protein biosynthesis, showed a decrease (compared with the controls), which at 6 h after irradiation was lower by 22 ± 5.5% (controls, 13.169 ± 3563 c.p.m. $mg^{-1}$ protein; irradiated, 10.186 ± 2830 c.p.m. $mg^{-1}$ protein). At the 24 and 48 h after irradiation, we showed an increase in the collagenous protein biosynthesis, which in relative values was 32.8 ± 9% and 22.5 ± 6.4% respectively (24 h controls, 14.900 ± 1300 c.p.m. $mg^{-1}$ protein; irradiated, 19.787 ± 2176 c.p.m. $mg^{-1}$ protein; 48 h controls, 15.158 ± 1364 c.p.m. $mg^{-1}$ protein; irradiated, 18.492 ± 2219 c.p.m. $mg^{-1}$ protein) (Figure 2).

In order to test the hypothesis that the endothelial cells are more radiosensitive during the period of angiogenesis (up to day 9), we performed the same experiments with chick embryos on day 14 of their development (Figures 3 and 4).

Photographs of the area of the CAM lying directly under the control or the test disc in the same egg provided us with visual confirmation of the antiangiogenic effect of X-rays on the CAM system on days 9 and 14 of the chick embryo development, 24 h after irradiation (Figure 5a and b, Figure 6a and b).

*Effects of X-rays and L-NAME and/or L-arginine on angiogenesis in the CAM* in vivo

Based on data from our experiments, we have chosen as optimal time points to evaluate further the mechanism(s) of

**Figure 4** Effect of a single dose of X-rays (10 Gy) on the CAM, expressed as collagenous protein biosynthesis (CPB), on day 14. An increase in CPB is observed 24 and 48 h after irradiation, which does not reach a statistically significant level.

**Figure 2** Effect of a single dose of X-rays (10 Gy) on the CAM *in vivo*, expressed as collagenous protein biosynthesis (CPB) on day 9. An increase in CPB is observed 24 and 48 h after irradiation. Results are expressed as mean ± s.e.mean % of control and are compared by paired *t*-test.

**Figure 3** Effect of a single dose of X-rays (10 Gy) on the CAM vascular density on day 14. Results are expressed as mean ± s.e. of vascular density for the irradiated (■) and non-irradiated (▒) CAM preparations. The results are not statistically significant.

**Figure 5** Photographs showing the CAM vessels on day 9, before (**a**) and after (**b**) irradiation. The irradiated CAM preparations contain fewer vessels than the non-irradiated, at 24 h after irradiation (stereoscope, original magnification × 16).

action of X-rays the ninth day of chick embryo development, when the CAM shows its maximum angiogenic activity, the sixth hour after irradiation for measuring collagenous protein biosynthesis, and the 24th hour after irradiation for measuring vascular density. In order to test the hypothesis that the NO pathway is involved in the antiangiogenic effect of X-rays, we have tested substances involved in the NO synthesis in our system, i.e. L-NAME and L-arginine.

*Effects of L-NAME* The specific inhibitor of the inducible nitric oxide synthase (iNOS), L-NAME, causes a dose-dependent increase in vascular density and basal collagenous protein biosynthesis in the CAM system (Pipili-Synetos *et al.*, 1993, 1994), while the D-isomer of L-NAME, D-NAME, has no effect on angiogenesis in the CAM (Pipili-Synetos *et al.*, 1994).

By adding 3.3 μg of L-NAME per disc (11.7 nmol per disc) immediately after irradiation of the CAM on the ninth day of chick embryo development, we found that L-NAME almost completely reversed the antiangiogenic effects of X-rays, as measured by measuring vascular density and collagenous protein biosynthesis (Figures 7 and 8).

When we added L-NAME to our system 50 min before the irradiation procedure, we observed the same reversal phenomenon of the antiangiogenic effect of X rays ($35 \pm 4.8\%$ over the controls in vascular density). This increase was greater than that seen with L-NAME added immediately after irradiation. When L-NAME was replaced by its inactive enantiomer, D-NAME (3.3 μg per disc), we did not observe any angiogenic effect and no reversal of the irradiation-induced antiangiogenesis (Figures 5, 9 and 10).

A 3-fold increase in the concentration of L-NAME (11.7 μg per disc) on day 14 of chick embryo development had no significant effect on the vascular density and the collagenous protein biosynthesis.

**Figure 6** Photographs showing the CAM vessels on day 14, before (**a**) and after (**b**) irradiation. No differences are seen in the number of vessels (vascular density), 24 h after irradiation (stereoscope, original magnification × 16).

*Effects of L-arginine* In order to establish that the angiogenic effect of L-NAME was caused by inhibition of NO synthase, we attempted to reverse the effect of this agent

**Figure 7** Effect of irradiation (IR) alone (10 Gy), L-NAME or both treatments simultaneously (L-N + IR) on the CAM vascular density on day 9 and 24 h after irradiation. Results are expressed as mean ± s.e. as % of the control CAM mean, and are compared by paired *t*-test with the control CAM preparations ($P < 0.01$).

**Figure 8** Effect of irradiation (IR), L-NAME or both treatments simultaneously (IR + L-NAME) on the CAM CPB on day 9, 6 h after irradiation. Results are expressed as mean ± s.e. as % of the control CAM mean, and are compared by paired *t*-test with the control CAM preparations ($P < 0.01$).

**Figure 9** Effect of irradiation (IR), D-NAME or both treatments simultaneously (D-N + IR) on the CAM vascular density on day 9, 24 h after irradiation. Results are expressed as mean ± s.e. as % of the control CAM mean, and are compared by paired *t*-test with the control CAM preparations.

by L-arginine, the endogenous substrate for NO synthase (Palmer *et al.*, 1988).

The effects of L-arginine on the regulation of angiogenesis were studied on the CAM model *in vivo* by other investigators (Pipili-Synetos *et al.*, 1994). It was found that L-arginine caused a small, but significant, decrease in collagenous protein biosynthesis and a non-significant decrease in vascular density. When L-arginine was combined with L-NAME, the angiogenic effect of the latter was completely abolished and a small inhibition of angiogenesis was observed (Pipili-Synetos *et al.*, 1994).

L-arginine was added immediately after irradiation and the vascular density was assessed at 24 h after irradiation. L-arginine alone caused a small decrease in the number of vessels ($-11.3 \pm 4.9\%$, $n=12$, $P<0.05$), but did not influence the antiangiogenic effect of irradiation ($-44.7 \pm 2.9\%$, $P<0.001$). When L-arginine was combined with L-NAME, the angiogenic effect of L-NAME was completely abolished on the non-irradiated and irradiated CAM ($n=12$, $-3.7 \pm 4.2\%$, $P=0.37$ and $-6.8 \pm 6.4\%$, $P=0.097$ respectively). Figure 11 summarises our results on the effect of L-arginine at a dose of 12.2 μg per disc.

**Figure 10** Effect of irradiation (IR), D-NAME or both treatments simultaneously (D-N + IR) on the CAM CPB on day 9, 6 h after irradiation. Results are expressed as mean ± s.e. as % of the control CAM mean, and are compared by paired *t*-test with the control CAM preparations.

**Figure 11** Effect of L-arginine (L-ARG), L-arginine plus L-NAME (ARG + L-N), L-arginine plus irradiation (ARG + R) or L-arginine plus L-NAME plus irradiation (R + ARG + L-N) simultaneously on the CAM vascular density on day 9, 24 h after irradiation. Results are expressed as mean ± s.e. as % of the control CAM mean, and are compared by paired *t*-test with the control CAM preparations.

*Histological studies*

The results of the histological studies demonstrated that the irradiated CAM preparations contained significantly fewer stromal cells ($45 \pm 8$ per high-power field) and lower microvessel density ($19 \pm 2$ per 10 high-power fields) than the non-irradiated CAM preparations ($90 \pm 9$ stromal cells per high-power field and $30 \pm 2$ microvessel density per 10 high-power fields respectively). When CAM preparations were irradiated and immediately treated with L-NAME, the stromal cell number and microvessel density were not significantly different from those of the control CAM

**Figure 12** Photographs showing tissue sections of the CAM on day 9, 24 h after irradiation (**a**) before irradiation, (**b**) after irradiation and (**c**) after adding L-NAME immediately after irradiation. The irradiated CAM preparation contains fewer vessels than the non-irradiated, whereas the CAM preparations that received irradiation plus L-NAME contain more vessels and stromal cells than the irradiated preparation (haematoxylin–eosin).

preparations (99±8 stromal cells per high-power field, microvessel density 32±2 per 10 high-power fields). At 6 h after irradiation, we observed gaps between endothelial cells, oedema of the intercellular space and thrombi in the vessels (Figure 12).

### Discussion

In the present study, it was shown that, in the *in vivo* CAM system, X-rays mediate their antiangiogenic effects through an action on NO synthase. Many studies published in recent years have shown the effect of radiation on endothelial cells *in vitro* and *in vivo*. Some studies have investigated the effect of radiation on the survival of endothelial cells using various *in vitro* models. The cell survival curves generated from these studies use the multihit model to explain basic radiobiological parameters (Hall, 1994). These *in vitro* models have shown that endothelial cells have moderate radiosensitivity compared with other cell types, such as fibroblasts, and that survival is dose and cell cycle dependent (Degowin *et al.*, 1976; Rhee *et al.*, 1986; Rubin *et al.*, 1989). Vegt *et al.* (1985) have shown that irradiation decreases sodium-dependent transport by impairment of the transport unit and has opposite effects on membrane-bound enzyme activity. Another more recently published study, using an *in vitro* model, demonstrated that the sensitivity of endothelial cells to radiation is affected by the microenvironmental conditions under which experiment is carried out (Fuks *et al.*, 1992).

*In vivo* models have also been used to measure endothelial cell survival. Ward *et al.* (1985) have found in the rat lung after irradiation of the hemithorax *in vivo* that the dose–effect curves for arterial perfusion, endothelial dysfunction and interstitial fibrosis exhibit similar, but not identical, radiosensitivities. Stewart *et al.* (1995) studied the blood–spinal cord barrier function and morphometry in rat spinal cord and speculated that the observed decrease in endothelial cell density was probably a direct result of irradiation. They speculated that the vascular bed has two possible responses to endothelial loss. Firstly, the remaining endothelial cells may elongate and send out cytoplasmic processes to fill in the gaps in the vessel walls, followed by endothelial proliferation to restore normal cell density, and secondly, the affected vascular segments may become permanently occluded, and new vesels may form to take their place.

Data obtained from the clinic have shown that some of the major functions of blood vessels are grossly affected by irradiation; for example, blood coagulation and thrombolysis, through the release of von Willebrand factor from endothelial cells, enhanced production of prostacyclins and suppression of the effects of plasminogen activator. The extracellular matrix profoundly influences the response of endothelial cells to radiation, either directly or indirectly (Sporn *et al.*, 1984; Mori *et al.*, 1991; Raymond *et al.*, 1990; Ornitz *et al.*, 1995). The endothelial cell is believed to be the most radiosensitive component of the vascular wall, as determined by ultrastructural studies. During the acute phase, some endothelial cells are killed and mural thrombi form to narrow or obliterate the vessel lumen. A few hours to a few weeks after moderate doses of radiation, there is an increased permeability of the capillary wall, as manifested by the associated oedema. As might be expected, the rapidly proliferating endothelial cells of newly developing capillaries are more sensitive to radiation than endothelial cells in older capillaries (Gillette *et al.*, 1985). One of the most constant early alterations seen in the capillaries and prearterioles after irradiation is dilation of the vessel. This can be accompanied by endothelial cell swelling, degeneration, necrosis and cellular inflammatory infiltrate. Increased vascular permeability with resulting tissue oedema is a common early manifestation.

During the chronic phase, the basement membrane of the capillary wall is thickened, and this is presumed to contribute to decreased capillary permeability. Still later, the number of small vessels is decreased through the process of vessel occlusion. The most frequent changes in vessels of medium and small calibre, particularly arteries, occur in the intima. These are manifested by swelling and vacuolation of the endothelial cells. The sequelae of small blood vessel obliteration vary with the organ in question (Byhardt and Moss, 1989).

Another important aspect regarding the involvement of the NO pathway in angiogenesis is in the pathogenesis of ultraviolet light B-induced vasodilatation of the microcirculation of rat skin *in vivo*. This response was abolished by NOS inhibitors and the effectiveness of canavanine implies that the inducible form of NO synthase is involved. Indomethacin was as effective as the NOS inhibitors, suggesting a link bewtween vasodilator prostaglandins and nitric oxide within the microcirculation (Warren *et al.*, 1993; Deliconstantinos *et al.*, 1995).

For the first time, we have used the CAM vessel to study the effects of ionising radiation on angiogenesis. In the system used, our results have shown that on the ninth day of chick embryo development the antiangiogenic effects of X-rays were more profound than those observed on day 14. This is mainly because the endothelial cells of the CAM vasculature are more sensitive to irradiation on the ninth day, as on this day the CAM resembles actively growing tissues regarding cell and vessel proliferation rates. This is in agreement with the results of Gillette *et al.* (1985) in a different *in vivo* system and with the findings of Wang *et al.* (1995), who have observed that the effect of radiation on the expression of an antigen present in tissues undergoing angiogenesis is greater in semi-confluent (proliferating) compared with confluent (non-proliferating) endothelial cells.

By measuring the vascular density and the collagenous protein biosynthesis, 24 and 6 h after irradiation, respectively, we found that the angiogenesis started at a faster rate than in the control CAM. These results can be explained on the basis of our observations from the histological sections showing a large number of thrombosed vessels, since it is known that microthromboses accelerate angiogenesis (Baker and Krochak, 1989; Fajardo and Berthrong, 1982; Tsopanoglou *et al.*, 1993). The temporal difference between the vascular density and the collagenous protein biosynthesis is probably due, firstly, to the accelerated non-vascular collagenous protein biosynthesis during the active repair process of the CAM, as it is known that collagenous proteins represent 80% of the total basement membrane proteins formed by the CAM (Maragoudakis *et al.*, 1988), and secondly, to the fact that the effect of irradiation on endothelial cells is a complex process, as mentioned above.

To test our initial hypothesis that NO is involved in the antiangiogenic effect of irradiation on the CAM, we used L-NAME, a specific NO synthase inhibitor (Moncada *et al.*, 1993). L-NAME, but not D-NAME, significantly enhanced both the vascular density and the CPB of the non-irradiated CAM, suggesting a negative regulatory role for the endogenous NO on CAM angiogenesis (Pipili-Synetos *et al.*, 1994). The angiogenic effect of L-NAME may be underestimated here, if the vasoconstrictive properties of L-NAME are maintained for 24 h and they affect the vascular density determination negatively. L-arginine had a modest angiogenic effect on the non-irradiated CAM, apparently by increasing the endogenous NO synthesis. This modest antiangiogenic effect may be a result of the reduced ability of exogenous arginine to compete with the endogenous one. The effect of L-arginine was further validated when L-arginine completely abolished the angiogenic effect of L-NAME on the irradiated or no-irradiated CAM. Furthermore, L-NAME completely reversed the irradiation-induced antiangiogenic effect on the CAM, as determined by means of both vascular density and CPB. The two phenomena quantitatively paralleled each other closely. These reversal effects were stereospecific for L-NAME, and they were completely abolished by L-arginine, suggesting that NO synthase activity plays an important role in the irradiation-induced antiangiogenic effect of the actively proliferating CAM. The above reversal effects by L-NAME were further corroborated by histological evidence which also extended the L-NAME protective effect to the irradiation-induced cytotoxic effects on CAM stromal cells. The density of these cells is apparently reduced by irradiation owing to oedema and apoptosis (Baker and Krochak, 1989; Lichter and Lawrence, 1995). In general, the histological results of the CAM treatment with either irradiation, L-NAME or the simultaneous treatments of irradiation and L-NAME parallel the present vascular density and CPB data.

Our results suggest that irradiation enhances NO synthesis in the actively proliferating CAM, resulting in inhibition of angiogenesis, and cytotoxicity. These are in agreement with the findings of Voevodskaya and Vanin (1992), who have shown on a different *in vivo* system that gamma-irradiation of mice at a sublethal dose of 7 Gy enhanced the formation of NO in the liver, intestine, lung, kidney, brain, spleen or heart of the animals. Recent studies support the notion that NO mediates vasodilator-induced angiogenesis in rabbit corneas (Ziche *et al.*, 1994), and angiogenesis induced by monocytes after stimulation with liposaccharide (Leibovich *et al.*, 1994). These results are in contrast with the present data, and other recent studies (Papaioannou *et al.*, 1995; Pipili-Synetos *et al.*, 1994) on actively proliferating CAM. The difference may be due to the fact that the former studies (Ziche *et al.*, 1994; Leibovich *et al.*, 1994) were conducted on adult and highly differentiated tissues compared with the embryonic actively proliferating CAM. Other differences may be important for understanding this controversy. The CAM is a relatively simpler system compared with the more complex systems stimulated by exogenous vasodilators or inflammators (e.g. substance P or liposaccharide). Vasodilation may contribute to the observed angiogenicity of NO by making capillaries more visible. Inflammation would generate NO and growth factors, the observed angiogenesis resulting from the latter factors rather than NO. NO synthase inhibitors would reduce NO synthesis and NO-induced inflammation, but could result in inhibition of angiogenesis under certain conditions of NO-induced and exogenous stimulant-induced inflammation, especially under chronic conditions (e.g. 5–15 days for the corneal bioassay), when tissue repair may complicate angiogenesis determinations. The cortisone used in the present study does not effect CAM angiogenesis and apparently reduces inflammation (Folkman *et al.*, 1983; Pipili-Synetos *et al.*, 1994), so that the irradiation-induced NO inhibits CAM angiogenesis in the short term with minimal interference by CAM inflammation or repair. Overall, it appears that angiogenesis can be complicated by the experimental conditions, especially the concentration of released NO and growth factors, inflammation, the kinetics of tissue repair and the cell proliferation rate.

Although no evidence is presented here regarding the molecular mechanisms of the antiangiogenic and cytotoxic effect of NO on the irradiated CAM, it is hypothesised that the radiation-induced NO interacted with superoxide anion radicals ($\cdot O_2^-$) for the synthesis of the cytotoxic peroxynitrite anion ($ONOO^-$), which caused stromal endothelial and other tissue injury, including inhibition of angiogenesis. This hypothesis is in agreement with recent work on peroxynitrite-induced injury to pulmonary surfactants (Haddad *et al.*, 1993) and on the role of NO in cellular redox reactions (Dreher and Junod, 1996).

In conclusion, the irradiation-induced antiangiogenic and cytotoxic effects of X-rays observed on the actively proliferating CAM appear to be NO dependent. This proposed mechanism may offer a new insight into the series of events involved in pathological angiogenesis and into the mechanisms of action of X-rays as well. Further work on experimental tumours in animals is in progress to evaluate these parameters in other *in vivo* systems.

### References

AMEZCUA JL, PALMER RMJ, DE SOUZA BM AND MONCADA S. (1989). Nitric oxide synthesized from L-arginine regulates vascular tone in the coronary circulation of the rabbit. *Br. J. Pharmacol*, **97**, 1119–1124.

BAKER DG AND KROCHAK RJ. (1989). The response of the microvascular systems to radiation: a review. *Cancer Invest.*, **7**, 287–294.

BYHARDT RW AND MOSS WT. (1989). The heart and blood vessels. In *Radiation Oncology – Rationale, Technique, Results.* Moss WT and Cox JD (eds) pp. 277–284. The CV Mosby Company: St Louis, USA.

DEGOWIN RL, LEWIS LJ, MASON RE, BORKE MK AND HOAK JC. (1976). Radiation-induced inhibition of human endothelial cells replicating in culture. *Radiat. Res.*, **68**, 244–250.

DELICONSTANTINOS G, VILLIOTOU V AND STAVRIDES JC. (1995). Release by ultraviolet B (uvB) radiation of nitric oxide (NO) from human keratinocytes: a potential role for nitric oxide in erythema production. *Br. J. Pharmacol.*, **114**, 1257–1265.

DREHER D AND JUNOD AF. (1996). Role of oxygen free radicals in cancer development. *Eur. J. Cancer*, **32A**, 30–38.

FAJARDO LF AND BERTHRONG M. (1982). Vascular lesions following radiation. *Pathol. Ann.*, **23**, 297–330.

FEELISCH M AND NOACK EA. (1987). Correlation between nitric oxide formation during degradation of organic nitrates and activation of guanylate cyclase. *Eur. J. Pharmacol.*, **139**, 19–30.

FOLKMAN J, LANGER R, LINHARDT RJ, HAUDENSCHILD C AND TAYLOR S. (1983). Angiogenesis inhibition and tumour regression caused by heparin or a heparin fragment in the presence of cortisone. *Science*, **221**, 719–725.

FOLKMAN J. (1985). Tumor angiogenesis. *Adv. Cancer Res.*, **43**, 175–203.

FOLKMAN J. (1995). Angiogenesis in cancer, vascular, rheumatoid and other disease. *Nature Med.*, **1**, 27–31.

FUKS Z, VLODAVSKY I, ANDREEFF M, MCLAOUGHLIN M, HAIMOVITZ-FRIEDMAN A. (1992). Effects of extracellular matrix on response of endothelial cells to radiation *in vitro*. *Eur. J. Cancer*, **28A**, 725–731.

GARG UC AND HASSID A. (1989). Inhibition of rat messangial cell mitogenesis by nitric oxide-generating vasodilators. *Am. J. Physiol.*, **257**, F60–F66.

GILLETTE EL, MCCHESNEY SL AND HOOPES PJ. (1985). Isoeffect curves for radiation-induced cardiomyopathy in the dog. *In. J. Radiat. Oncol. Biol. Phys.*, **11**, 2091–2097.

HADDAD IY, ISCHIROPOULOS H, HOLM BA, BECKMAN JS, BAKER JR AND MATALON S. (1993). Mechanisms of peroxynitrite-induced injury to pulmonary surfactants. *Am. J. Physiol.*, **265**, 555–564.

HALL EJ. (1994). *Radiobiology for the Radiologist.* Lippincott: Philadelphia, USA.

HARRIS-HOOKER SA, GAJDUSEC CM, WIGHT TN AND SCHWARTZ SM. (1993). Neovascular responses induced by cultured aortic endothelial cells. *J. Cell Physiol.*, **114**, 302–310.

HIBBS JB, TAINTOR RR, VAVRIN Z AND RACHLIN EM. (1988). Nitric oxide: A cytotoxic activated macrophage effector molecule. *Biochem. Biophys. Res. Commun.*, **157**, 87–94.

LEIBOVICH SJ, POLVERINI PJ, FONG TW, HARLOW LA AND KOCH AE. (1994). Production of angiogenic activity by human monocytes requires an L-arginine/nitric oxide-synthase-dependent effector mechanism. *Proc. Natl Acad. Sci. USA*, **91**, 4190–4194.

LICHTER AS AND LAWRENCE TS. (1995). Recent advances in radiation oncology. *N. Engl. J. Med.*, **6**, 371–379.

LUTZ W. (1978). Statistical methods as applied to immunological data. In *Handbook of Experimental Immunology*. Weir DM (ed.) pp. 1–29. Blackwell: Oxford.

MARAGOUDAKIS ME, PANOUTSACOPOULOU M AND SARMONIKA M. (1988). Rate of basement membrane biosynthesis as an index to angiogenesis. *Tissue Cell*, **20**, 531–539.

MISSIRLIS E, KARAKIULAKIS G AND MARAGOUDAKIS M. (1990). Angiogenesis is associated with collagenous protein synthesis and degradation in the chick chorioallantoic membrane. *Tissue Cell*, **22**, 419–426.

MONCADA S AND HIGGS A. (1993). The L-arginine–nitric oxide pathway. *N. Engl. J. Med.*, **329**, 2002–2010.

MORI S, TANAKA R AND MINAKAWA T. (1991). Effects of radiation on capillary endothelial cells derived from Mongolian gerbil brain. *Neurosurgery*, **29**, 658–662.

ORNITZ DM, HERR AB, NILSSON M, WESTMAN J, SVAHN CM AND WAKSMAN G. (1995). FGF-binding and FGF-receptor activation by synthetic heparin-derived di- and tri-saccharides. *Science*, **268**, 432–436.

PALMER RMJ, ASHTON DS AND MONCADA S. (1988). Vascular and endothelial cells synthesize nitric oxide from L-arginine. *Nature*, **333**, 664–666.

PAPAIOANNOU SE, HATJICONTI O, KATSAROU C, RAVAZOULA P, DIMOPOULOS J AND MARAGOUDAKIS M. (1995). Inhibition of nitric oxide synthase reverses the antiangiogenic effect of X-ray irradiation on chick embryo choriallantoic membrane. In *Vascular Endothelium: Responses to Injury*. Catravas J, Callow A and Ryan U (eds) pp. 306–308. Plenum Press: New York.

PIPILI-SYNETOS E, SAKKOULA E, HARALABOPOULOS G, ANDRIOPOULOU P, PERISTERIS P AND MARAGOUDAKIS ME. (1994). Evidence that nitric oxide is an endogenous antiangiogenic mediator. *Br. J. Pharmacol.*, **111**, 894–902.

PIPILI-SYNETOS E, PAPAGEORGIOU A, SAKKOULA E, SOTIROPOULOU G, FOTSIS G, KARAKIULAKIS G AND MARAGOUDAKIS M. (1995). Inhibition of angiogenesis, tumour growth and metastasis by the NO-releasing vasodilators, isosorbide mononitrate and dinitrate. *Br. J. Pharmacol.*, **116**, 1829–1834.

PRICE KM, HORNER KA AND MCNALLY NJ. (1992). Interaction of hydrogen peroxide and ionizing-radiation-induced damage. In *Radiation Science of Molecules Mice and Men*. Denecamp J and Hirst DG (eds) pp. 28–31. *Br. J. Radiol.* (suppl.).

PRIONAS SD, KOWALSKI J, FAJARDO LF, KAPLAN I, KWAN HH AND ALLISON AC. (1990). Effects of X-irradiation on angiogenesis. *Radiat. Res.*, **124**, 43–49.

RADOMSKI MW, PALMER RMJ AND MONCADA S. (1990). An L-arginine/nitric oxide pathway present in human platelets regulates aggregation. *Proc. Natl Acad. Sci. USA*, **87**, 5193–5197.

RAYMOND J, YOON SC AND TS'AO EH. (1990). Stimulation of radiation-impaired plasminogen-activator release by phorbol ester in aortic endothelial cells. *Proc. Soc. Exp. Biol. Med.*, **195**, 213–217.

RAVAZOULA P, HATJIKONTI O, MARAGOUDAKIS M AND PAPAIOANNOU SE. (1995). Histological and immunohistochemical evidence that L-NAME protects CAM from X-ray-irradiation-associated tissue injury. In *Vascular Endothelium: Response to Injury*. Catravas J, Callow A and Ryan U (eds) pp. 317–318. Plenum Press: New York.

RHEE JG, LEE I AND SONG CW. (1986). The clonogenic response of bovine aortic endothelial cells in culture to radiation. *Radiat. Res.*, **106**, 182–189.

RUBIN DB, DRAB EA AND BAUER KD. (1989). Endothelial cell subpopulations *in vitro*: cell volume, cell cycle and radiosensitivity. *J. Appl. Physiol.*, **67**, 1585–1590.

SCHEFLER WC. (1969). *Statistics in Biological Sciences*. Addison-Wesley: Reading, MA.

SCHMIDT HHHW, NAU H, WITTFOHT W, GERLACH J, PRESCHER K, KLEIN MM, NITROOMAND F AND BOHME E. (1988). Arginine is a physiological precursor of endothelium-derived nitric oxide. *Eur. J. Pharmacol.*, **154**, 213–216.

SIAMBLIS D, KARNABATIDIS D, HATJIKONDI O, KALOGEROPOULOU CH, KARDAMAKIS D AND DIMOPOULOS J. (1996). A novel radiological approach for the experimental study of angiogenesis: angiography of the chick embryo and its chorioallantoic membrane. *Eur. J. Radiol.*, **21**, 220–224.

SYNDER SH AND BREDT DS. (1992). Biological roles of nitric oxide. *Sci. Am.*, 28–35.

SPORN LA, RUBIN P, MARDER VJ AND WAGNER DD. (1984). Irradiation induces release of von Willebrand protein from endothelial cells in culture. *Blood*, **64**, 567–570.

STEWART PA, VINTERS HV AND WONG CS. (1995). Blood–spinal cord barrier function and morphometry after single doses of X-rays in rat spinal cord. *Int. J. Radiat. Oncol. Biol. Phys.*, **32**, 703–711.

TSOPANOGLOU NE, PIIPILI-SYNETOS E AND MARAGOUDAKIS M. (1993). Thrombin promotes angiogenesis by a mechanism independent of fibrin formation. *Am. J. Physiol.*, **264**, 1302–1307.

VEGT GB, WASSENAAR AM, KAWILARANG-DE HAAS EWM, SCHUTTE PP, VAN DER LINDEN M, DI BON-DE RUIJTER M AND BOON A. (1985). Radiation-induced changes in the cell membrane of cultured human endothelial cells. *Radiat. Res.*, **104**, 317–328.

VOEVODSKAYA NV AND VANIN AF. (1992). Gamma-irradiation potentiates L-arginine dependent nitric oxide formation in mice. *Biochem. Biophys. Res. Commun.*, **186**, 1423–1428.

WANG J-M, KUMAR S, VAN AGTHOVEN A, KUMAR P, PYE D AND HUNTER RD. (1995). Irradiation induces up-regulation of E9 protein (CD105) in human vascular endothelial cells. *Int. J. Cancer*, **62**, 791–796.

WARD WF, MOLTENI A, SOLLIDAY NH AND JONE GE. (1985). The relationship between endothelial dysfunction and collagen accumulation in irradiated rat lung. *Int. J. Radiat. Oncol. Biol. Phys.*, **11**, 1985–1990.

WARREN UB, LOI RK AND COUGHLAN ML. (1993). Involvement of nitric oxide synthase in the delayed vasodilation response to ultraviolet light irradiation of rat skin *in vivo*. *Br. J. Pharmacol.*, **109**, 802–806.

ZICHE M, MORBIDELLI L, MASINI E, AMERINI S, GRANGER HJ, MAGGI CA, GEPPETTI P AND LEDDA F. (1994). Nitric oxide mediates angiogenesis *in vivo* and endothelial cell growth and migration *in vitro* promoted by substance P. *J. Clin. Invest.*, **94**, 2036–2044.